# Essential oil extraction from clove, characterization and application

**DOI:** 10.1186/s13065-025-01685-x

**Published:** 2025-12-17

**Authors:** Gargi Ghoshal, Tenzin Dolker, Sakshi Gupta

**Affiliations:** Dr S. S. Bhatnagar University Institute of Chemical Engineering & Technology, Panjab University, Chandigarh, 160014 India

**Keywords:** Eugenol, Gelatin, Antioxidant, Antimicrobial, Active packaging material

## Abstract

Eugenol, a beneficial nutraceuticals majorly (80–90%) found in clove, has diverse applications due to its antimicrobial and antioxidant properties. Clove is quickly engrossed by diverse organs to metabolize in the liver. To prevent premature absorption and improved activity, water solubility, encapsulation of eugenol is the best solution. Maceration and Soxhlet were performed using ethanol, methanol and water as a solvents. Extracted clove essential oil were successfully encapsulated in gelatin hydrogel. The Soxhlet extraction method had the highest yield (44 ± 0.023) as compared to maceration. Encapsulation efficiency of Clove essential oil in gelatin hydrogel was 84.65 ± 0.12%. Characterization of hydrogel was done using FTIR, SEM and XRD and inclusion of Clove essential oil in hydrogel was established. The radical scavenging activity of Clove essential oil loaded hydrogel enhanced by 57%. (17 ± 0.3) mm and (15 ± 0.2) mm the zone of inhibitions were expressed in terms of gram negative bacteria *E. coli*. and gram positive bacteria *S. aureus.* When Clove essential oil incorporated hydrogel was coated on apples via the methods like dipping, brushing, coating was able to enhance the shelf life of fruits as active food packaging material.

## Highlights

- The research on Natural Hydrogels has attracted a significant attention as biological material.
- Edible hydrogel coatings were formulated with gelatin and clove extract.
- Eugenol extracted from clove has diverse applications.
- Clove essential oil (eugenol) was active in inhibiting the growth of gram negative bacteria *E. coli.*
- Gelatin hydrogels loaded with eugenol successfully helped in increasing the shelf life of apple.

## Introduction

Native to the north Maluka Islands of Indonesia *Syzygium aromaticum* (Clove) is widely used as a spice due to its medicinal, culinary and flavouring properties. Clove buds consist of carbohydrates, proteins, fats with volatile and non volatile fatty acids, cellulose, pentosans, resins, tannins, and mineral components [7, 20]. Cloves have antimicrobial, antioxidant, antiseptic, anti-inflammatory, carminative, local anaesthetic, warming and soothing properties. Clove essential oil contains a large amount of phenolic compounds, the main bioactive component being (80–90%) eugenol [29]. In medicine it is very valuable components as stimuli and its aromatic flavour and its carminative nature makes it precious. Clove essential oil is antiseptic, antibacterial and antibiotic in nature thus it is used in medicine, especially in oral cavity and pharyngeal treatments in dental problems, toothpastes, mouthwashes, dental creams, throat sprays and more to cleanse bacteria and to reduce pain in painful gums and improve by and large oral health [45].

Eugenol is an aromatic alcohol, belongs to phenol group. It is usually extracted from natural essential oils of *Lamiaceae*, *Myrtaceae* and *Myristicaceae* families plant leaves, buds, flowers etc. Eugenol is the most important component (80–90%) of clove essential oil [43]. Clove essential oil has colourless to light yellow color pure liquid, oily appearance and has spicy odour. Clove essential oil is slightly miscible in aqueous media, but organic solvents soluble extremely well. Due to its rapid absorption in diverse organs and metabolization in liver, to prevent premature absorption, Clove essential oil encapsulation is the best technique to enhance water solubility eventually better bioavailability. Due to its numerous properties, Clove essential oil has found diverse applications in various industries such as pharmaceutical, food and cosmetics [46]. Eugenol has a variety of nutraceutical properties, antifungal, anti-inflammatory and anti platelet. It also has good antioxidant properties, mainly due to the hydroxyl groups present within its structure, thus it scavenges free radicals and hamper the development of ROS [4, 34].

Eugenol has excellent antibacterial features against numerous species e.g. *S. aureus* and *E. coli* making it suitable for bioactive agent in active food packaging [6]. Eugenol is a hydrophobic molecule that can simply enter inside the lipo-polysaccharide cell membrane and penetrate the cytoplasm, and is thought to prevent Gram-negative bacteria damaging the cell membrane. When present inside cells, they can cause changes in cell structure and cause leakage of intracellular components. It improves hydrophobicity of active packaging, thus can be used in food preservation [28, 30, 31].

The novelty of this study includes the use of gelatin as a gelling agent and clove essential oil encapsulated within it to form a hydrogel. Encapsulation of clove essential oil improved the sensory properties of apples when used as an effective coating material for long-term storage [2]. The coated products confirmed better storage strength contrast to the control [12, 13]. Additionally detailed studies of hydrogel were achieved by evaluating its morphology, chemical bonding, antibacterial, antioxidant, and in vitro release properties. Specific objectives include: (1) Optimization of Clove essential oil extraction from cloves by maceration and Soxhlet method and its characterization. (2) Optimization of Clove essential oil loading in gelatin hydrogels and its characterization, application in fruits coating.

## Materials and methods

### Materials

Commercially accessible clove buds and apple were purchased from the local market of Panjab University, Chandigarh, India. Gelatin was obtained from Thermo Fisher scientific. Tween 80 and span 80 were supplied from (Rankem, Gurgaon). DPPH, pepsin (3000 U/g), pancreatin (100 U/mg) from Sisco Research Laboratories Private Limited (Mumbai, India), Methanol from Merck Specialities Private Limited (Mumbai, India), HCL from Loba Chemie Private Limited (Mumbai, India) were purchased. Potassium dihydrogen orthophosphate and di-Potassium hydrogen orthophosphate were purchased from SD Fine-chem Limited (Mumbai, India). All the chemicals were of analytical grades. Bacterial strains were purchased from IMTECH, Chandigarh.

### Techniques for clove essential oil extraction from clove

#### Maceration

The Clove essential oil extraction was done using maceration technique following the method described by Frohlich et al. [10]. The clove buds were collected and washed. After Sun drying, the clove buds were ground into a fine powder using mixer grinder. It was then stored in an airtight container. 10 g dried powder was immersed in 35mL distilled water. It was stirred well with the spatula, and kept still for 5 days by making the top of the beaker airtight. After each 24 h, the extracted liquid was placed in another beaker and was made airtight on top by using aluminium foil paper. Once 5 days were completed, the extracted liquid was then lyophilized and the final product as powder was received. Maceration was done first with water and then with ethanol and methanol. Extraction of Clove essential oil was done in triplicate from clove buds [10].

#### Soxhlet extraction

The Clove essential oil from clove buds was extracted in Soxhlet apparatus using solvents like methanol, ethanol according to Shafira et al. [39]. In a traditional solvent extraction practice of eugenol, clove buds were collected and washed, the buds were ground to powder using mixer grinder and kept in a dry place in airtight plastic container and was filled in a thimble and placed in Soxhlet apparatus for extraction. Extraction was done for 8 h at 70̊ C.

### Encapsulation of clove essential oil into hydrogel

The hydrogel was made according to Wan et al. [47] with slight modifications. To make hydrogels, the emulsions were prepared initially. Various concentrations of Clove essential oil extracted using Soxhlet apparatus and ethanol as a solvent (0, 0.5 and 1 mg/ml) were mixed with 2 g surfactant i.e., Span 80 and Tween 80 in the ratio of 1:19. Deionized water was added until the final sample weight was 20 g and the above mixture was continuously stirred using magnetic stirrer (IKA RET control-visc) for 15 min at 40 °C and 600 rpm. Gelatin solution was further prepared as; 0.75 g of gelatin was mixed with 10 mL deionized water at 80 °C, 20 min and 600 rpm. The emulsion prepared earlier, and gelatin solution was then taken in a ratio 3:2 and was stirred in magnetic stirrer at 600 rpm until hydrogel was formed. The hydrogel was kept in a refrigerator at 4 °C for 24 h prior to analysis. The process was repeated in triplicate [15].

### Encapsulation efficiency (EE)

The EE of Clove essential oil in the hydrogel was determined according to Li et al. [30] with some alteration. Clove essential oil was extracted using ethanol. Briefly, a certain amount of 0.5 g and 1.0 g Clove essential oil incorporated gelatin hydrogel was taken in separate beakers. 100 g samples were mixed with ethanol and homogenized at 39 °C for 10 min. After filtration, filtrate was collected. The quantity of Clove essential oil in supernatant was calculated using UV absorbance spectroscopy at the wavelength of maximum absorbance (λ_max_) 282 nm according to the calibration curve of the Clove essential oil -ethanol solution. The process was carried out in triplicate. Encapsulation efficiency of Clove essential oil was calculated by the formula:

1 $$ {\text{EE~\% }} = {\text{~}}\frac{{\begin{array}{*{20}c} {{\text{Total~nutraceutical~}}} \\ {{\text{content}}} \\ \end{array} - \begin{array}{*{20}c} {{\text{Surface~nutraceutical~}}} \\ {{\text{content}}} \\ \end{array} {\text{~}}}}{{{\text{Total~nutraceutical~content}}}}{\text{~}} \times 100 $$

### Swelling property of hydrogel

Swelling feature of hydrogels was tested according to the method of Tong et al. [44] with some modifications. To check the extent of swelling of hydrogels containing 0.5 g and 1.0 g Clove essential oil, the hydrogel was weighed followed by dipped in deionized water. The hydrogels were taken out, from time to time, from water and the surface was lightly wiped out using filter paper to eliminate the surface water. The weight was recorded, and the process was repeated till no volume change. The swelling ratio was determined using the Eqs. 2,

2 $$\:\text{\%}\:\text{D}\text{e}\text{g}\text{r}\text{e}\text{e}\:\text{o}\text{f}\:\text{s}\text{w}\text{e}\text{l}\text{l}\text{i}\text{n}\text{g}\:\text{o}\text{r}\:\text{s}\text{w}\text{e}\text{l}\text{l}\text{i}\text{n}\text{g}\:\text{r}\text{a}\text{t}\text{i}\text{o}=\:\frac{{\text{W}}_{\text{S}}-{\text{W}}_{\text{D}}}{{\text{W}}_{\text{D}}}\:\times\:100$$

where; $\:{\text{W}}_{\text{S}\:\:}$ and $\:{\text{W}}_{\text{D}\:}$ are the weight of swollen hydrogel before and after drying.

### Fourier transform infrared spectroscopy (FTIR)

The FTIR spectrophotometer reading was taken to get information on molecular structure of hydrogels in the range 4000 cmˉ¹– 650 cmˉ¹ and also to investigate a comparative study of chemical bonding between different hydrogels [27].

### X- ray diffraction technique (XRD)

To investigate the crystallographic properties of the hydrogels, XRD was performed using Philips diffractometer equipped with a general area detector diffraction system in the diffraction angle ranging from 5° to 140° (2ϴ).

### Scanning electron microscope (SEM)

The morphological structure of the hydrogels was examined using a scanning electron microscope (JEOL JSM 6100) at 0.3 kV to 30 kV. A thin layer of platinum was used to coat the sample to improve the electrical conductivity of the surface. The magnification ranged from 500x to 2000x.

### Contact angle

Contact angle measurements of the hydrogels were performed to check the surface wettability expressed as the angle between the liquid interface and the solid surface. This step was implemented from the study [31]. In this study, a hydrogel was placed on a glass slide, a water droplet was dropped onto the horizontal surface of the hydrogel, and its contact angle was measured by Goniometer (Krüss, type G10, Netherlands).

### Color

The colour measurement of hydrogel was performed using Hunter Lab Spectrophotometer (Color Flex model, U.S.A). The colour values were measured in terms of L*, a* and b*. L* indicates darkness to lightness in 0 to 100 scale, +a* for red and -a* greenness and + b* for yellow to -b* blueness [39]. The analysis was done in triplicate.

The total colour difference (∆E) was calculated as follows [5]:

3 $$\Delta {\text{E }}=\surd \left[ {{{\left( {\Delta {\text{L}}} \right)}^2}+{{\left( {\Delta {\text{a}}} \right)}^2}+{{\left( {\Delta {\text{b}}} \right)}^2}} \right]$$

### Gas chromatography mass spectrometry (GCMS)

GCMS of Clove essential oil was performed in THERMO Triple Quadrupole MS (Trace 1300 GC coupled with THERMO- TSQ 8000), SAIF, PU. Existing components in the extract were identified by comparing their mass spectra against retention time [42].

### Antimicrobial activity of clove essential oil

Antimicrobial activity of clove essential oil was done using well diffusion method against gram negative bacteria *E. coli*. The antimicrobial activity test was performed according to the literature study of Khan et al. [26] with some modifications.

### Antioxidant analysis

The antioxidant properties of the hydrogels were studied using 1, 1 diphenyl 2 picrylhydrazyl (DPPH), according to the method of Permatananda et al. [38] with some modifications. The antioxidant properties of the hydrogels were calculated using the following formula:

4 $$\:\text{\%}\:\text{R}\text{a}\text{d}\text{i}\text{c}\text{a}\text{l}\:\text{s}\text{c}\text{a}\text{v}\text{e}\text{n}\text{g}\text{i}\text{n}\text{g}\:\text{a}\text{c}\text{t}\text{i}\text{v}\text{i}\text{t}\text{y}=\:\frac{{\text{A}\text{b}\text{s}}_{\text{c}\text{o}\text{n}\text{t}\text{r}\text{o}\text{l}}-{\text{A}\text{b}\text{s}}_{\text{s}\text{a}\text{m}\text{p}\text{l}\text{e}}}{{\text{A}\text{b}\text{s}}_{\text{c}\text{o}\text{n}\text{t}\text{r}\text{o}\text{l}}}\:\times\:100$$

where; $\:{\text{A}\text{b}\text{s}}_{\text{c}\text{o}\text{n}\text{t}\text{r}\text{o}\text{l}\:}\:$ and $\:{\text{A}\text{b}\text{s}}_{\text{s}\text{a}\text{m}\text{p}\text{l}\text{e}}$ are the absorbances of the control and sample.

### In vitro drug delivery

In-vitro release study of nutraceutical was carried out to study the control release of the eugenol in the gastrointestinal tract (GIT). Simulated gastric fluid (SGF) and simulated intestinal fluid (SIF) with pH values of 1.2 and 6.8 was made using modified method of Azad et al. [3]. 10% Clove essential oil was used in both the medium to make Clove essential oil soluble. At 10 min. time interval 1mL of sample was withdrawn and 1mL was added again in the same beaker. This test was conducted for 4 h, and the samples in the test tube were analysed using a UV-visible spectrophotometer to study drug delivery.

### Application of hydrogel coating

The fruit samples were coated with the help of two methods i.e. brushing and dipping. Five apple samples were taken for the experimental investigation to study the effectiveness of the coating of Clove essential oil loaded gelatin hydrogel as active food packaging material. The apples were washed with water, dried at room temperature and separated into three groups. Group A consists of the fruit samples are without coating, Group B consists of apples in which coating was applied in dipping method. In dipping method, the fruits were immersed in coating solution of hydrogel and held for 2 to 3 min then fruits were removed and dried at room temperature. In group C brushing method was applied on apple. After coating, the fruits were stored at 4 ± 1 °C for 15 days for their detailed study.

#### Weight loss

During the storage period, weight loss of the apples was evident. Weight loss was measured in a weighing balance and was expressed as percentage of the initial weight. The weight loss was checked for the apples by comparing the uncoated apple with the apples coated via brushing and dipping method on days 0, 2, 6, 9, 13 and 15th [47].

The weight loss % was calculated by the formula:

5 $$\:\text{W}\text{e}\text{i}\text{g}\text{h}\text{t}\:\text{l}\text{o}\text{s}\text{s}\:\text{\%}=\:\frac{{\text{W}}_{\text{I}}-{\text{W}}_{\text{F}}}{{\text{W}}_{\text{I}}}\:\times\:100$$

Where, $\:{\text{W}}_{\text{I}\:}$, $\:{\:\text{W}}_{\text{F}\:}$ are the initial and final weight of apple samples respectively during storage.

#### Sensory evaluation

Sensory analysis of coated apples was carried out till 15th day of storage. Sensory evaluation of the preserved apples was performed by 20 trained panelists. Our study was reviewed by an appropriate ethics committee and they have waived the need for informed consent. The written consent of our study adhered to the Declaration of Helsinki was taken prior testing from all the panelists to participate in sensory evaluation.

The sensory analysis was carried out by using a nine-point Hedonic scale (1 = most disliked attribute, 9 = most liked attribute) in Dr. S.S.BU.I.C.E.T, Panjab University, Chandigarh. Samples were coded with different codes and panellists were asked to rank scores. The panelists scored for various sensory characteristics such as color, odor, taste, flavor, texture, appearance, and overall acceptability [33].

#### Firmness

The firmness of coated and uncoated apple was determined by texture analyzer (TA. XT plus, Stable Microsystems, UK) with 5 mm cylindrical probe. The test was performed using 5 mm/s test speed; 5 g trigger load and two 5 mm compressions separated by a 5 s interval **(**Wang et al., [50].

### Statistical analysis

Mean ± standard deviation was used to represent the data. For data visualization origin Pro 8.5 was utilized. The statistical analysis involved examining the significance of variances in treatments among groups through the utilization of ANOVA, at significance level of 5%, using SPSS software. Each experiment was conducted 3 times.

## **Results and discussion**

### Evaluation of clove essential oil from clove

#### Comparison study of clove essential oil yield obtained from different solvents and different extraction method

The yield of Clove essential oil obtained after extraction from cloves using maceration and Soxhlet method is shown in Table 1.

**Table 1:** Comparison of yield with different solvents and extraction methods

Solvent used (Extraction method)	Clove essential oil yield obtained (%)
Water (Maceration)	16 ± 0.025
Ethanol (Maceration)	22 ± 0.062
Methanol (Maceration)	19 ± 0.078
Ethanol (Soxhlet)	44 ± 0.023
Methanol (Soxhlet)	38 ± 0.037

The results showed that Soxhlet method using ethanol as solvent had the highest yield (44 ± 0.023) % whereas the maceration process performed using water as solvent gave the lowest yield (16 ± 0.065) %. This result is consistent with the literature study by Adaramola and Onigbinde [1]. There, water maceration resulted in the lowest yield (15.1%). A literature study by Guan et al. [16] showed that the yield of Clove essential oil was obtained after Soxhlet extraction was 41.8%. The clove essential oil containing hydrogel was stable at 4̊ C till 15 days and the property of hydrogel was unchanged. After 15 days the hydrogel was hard and at room temperature after 7 days mould growth was observed.

#### FTIR spectroscopy analysis

**Fig. 1:**
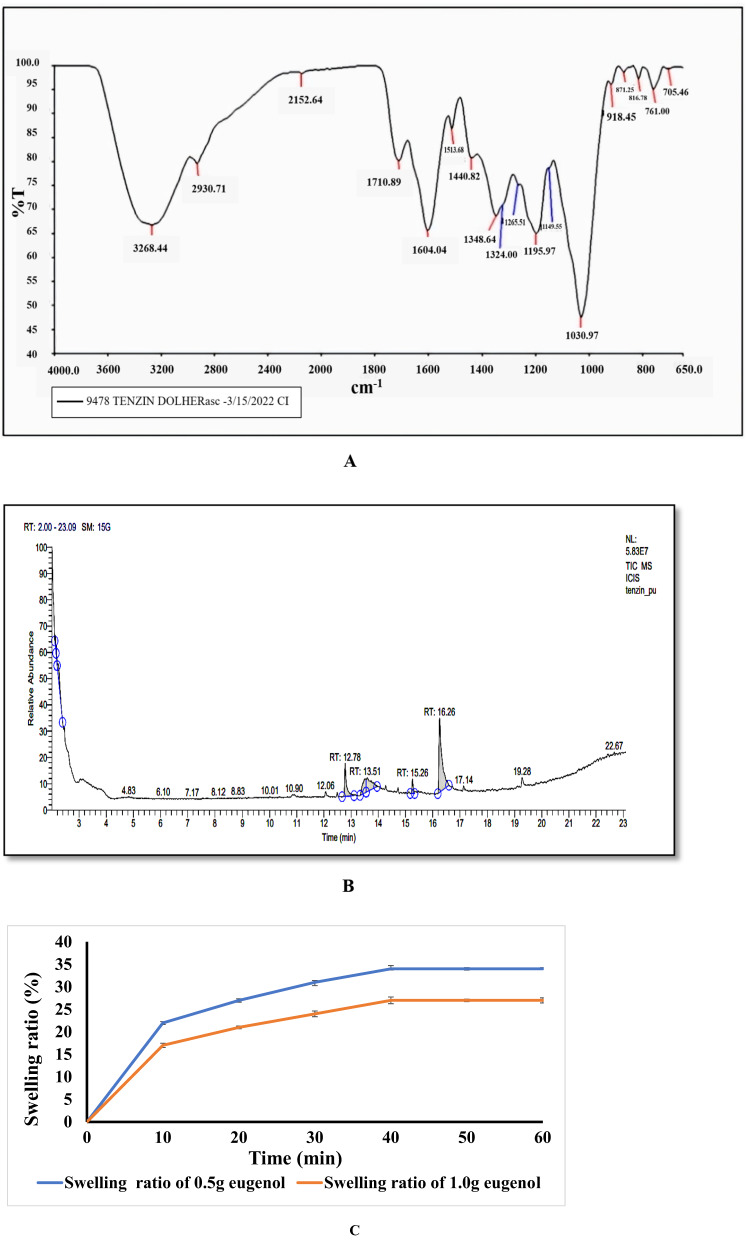

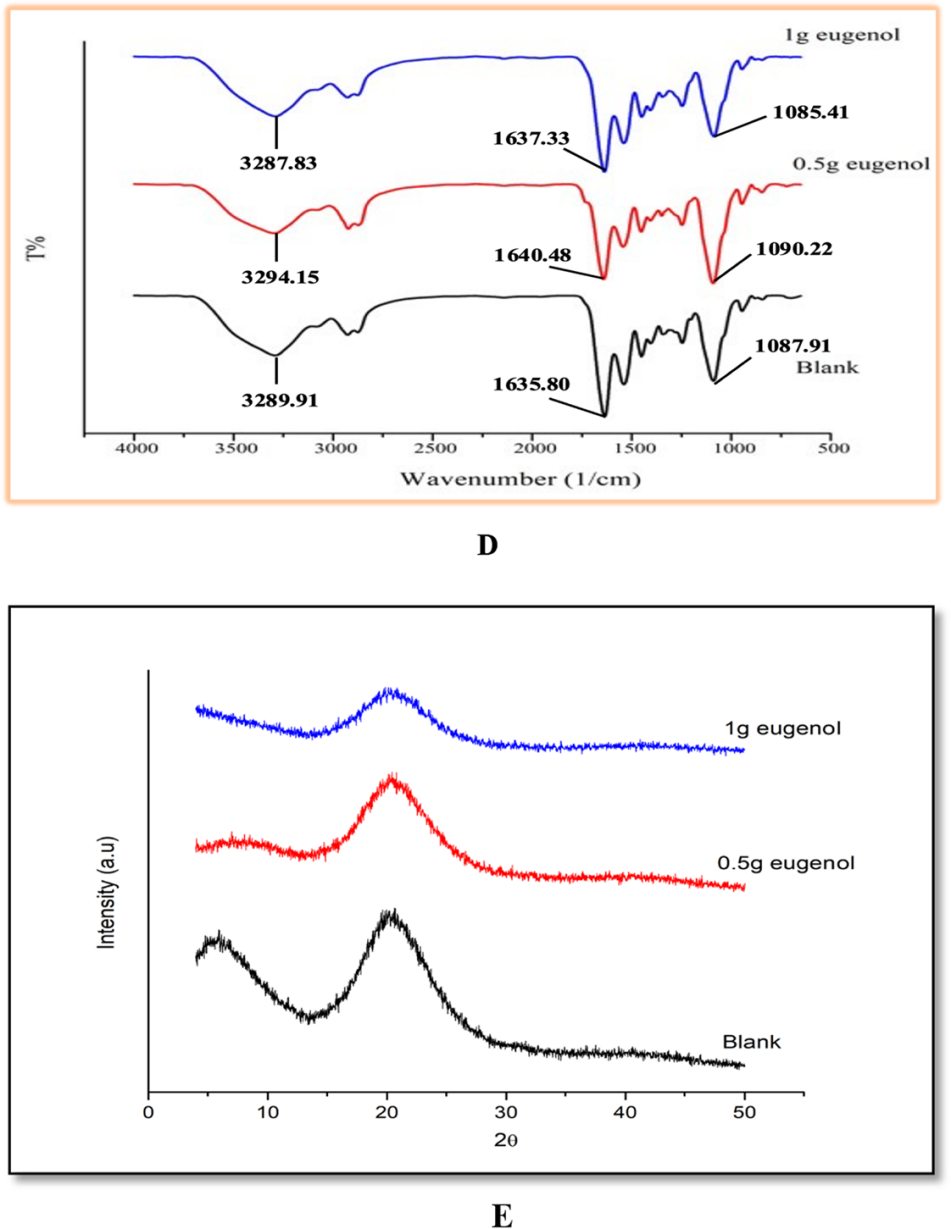
**A** FTIR spectra of eugenol. **B** GCMS of clove extract. **C** Graph of Swelling Ratio versus Time. **D** FTIR spectra of blank hydrogel, hydrogel with 0.5 g eugenol incorporation and hydrogel with 1.0 g eugenol incorporation. **E** XRD of blank and eugenol loaded hydrogel

Figure 1A shows the FTIR spectrum of Clove essential oil with a characteristic peak at 3268.44 cmˉ¹ (-O-H stretching bond). The peak at 2930.71 cmˉ¹ represents the symmetric -CH3 stretching, and the peak at 2152.64 cmˉ¹ represents the -C-H stretching bond.

Different peaks at 1710.89, 1604.04, 1513.68 and 1440.82 cmˉ¹ represent asymmetric --C-O stretching, asymmetric -CH3 stretching and symmetric -CH3 stretching. The peak at 1348.64 represents -O-H in plane bending, 1198.97cmˉ¹ is assigned to -C-C stretching, the peak at 1030.97 cmˉ¹ represents -C-O-C bending, 816.78 cmˉ¹ represents -C-C ring stretching, and 761.00 cmˉ¹ peak represents hydrogen bond bonded out of the plane. The present study on chemical bonds present in Clove essential oil was consistent in accordance with the study by Nguyen et al. [36].

#### GCMS of clove extract

GCMS analysis of clove extract showed four major peaks with different retention times and 11 components were identified as shown in Fig. 1(B). Components was identified by comparing the retention time and peak appearances of extracts. The four main peaks shown in Fig. 1B were identified as eugenol at retention time of 13.51 min, caryophyllene at 15.26 min, eugenol acetate at 16.26 min, and 2**-**Phenyl-5,6-dihydro-4 H-1,3-oxazine at 12.78 min. The main component of Clove essential oil was eugenol with 54.88%, followed by eugenol acetate with 19.46%. High concentration of Clove essential oil in clove enabled antibacterial and antioxidant properties [23].

The antimicrobial and antioxidant properties are mainly due to the phenolic OH groups of Clove essential oil, which scavenge free radicals and tend to destroy the cellular structure of bacterial cells and cause cell death. However, the small peaks shown in Fig. 1B are due to the other components which were present in the clove extract and are listed in Table 2.

**Table 2:** Components present in clove extract using GCMS study

S. No	Components	Retention time (min)	Peak area (%)
1	Eugenol acetate	16.26	19.46
2	Caryophyllene	15.26	4.48
3	Phenol, 2,4-bis(1,1-dimethylethyl)-	13.59	3.27
4	Pentanoic acid, 5-hydroxy-, 2,4-di-t-butylphenyl esters	13.59	2.49
5	Eugenol	13.51	54.88
6	2-Phenyl-5,6-dihydro-4 H-1,3-oxazine	12.78	6.65
7	Diethyl Phthalate	2.25	2.14
8	Phthalic acid, ethyl hex-2-yn-4-yl ester	2.25	2.07
9	3-fluoro-á,5-dihydroxy-N-methyl-Benzeneethanamine	2.25	1.82
10	N-Methyltaurine	2.17	1.54
11	(2-Aziridinylethyl)amine	2.17	1.20

In the current study, it was observed that the extraction method affected the peak areas of the components in clove extracts. The main components contained in the clove extract after Soxhlet extraction were eugenol and eugenol acetate [23]. The main components were eugenol and caryophyllene after hydro distillation extraction [42]. However, eugenol was found to be the main component of clove essential oil, with peak areas ranging from 50 to 60% of the total clove essential oil.

#### Antimicrobial activity analysis of clove extract

Clove essential oil was active in inhibiting the growth of gram negative bacteria *E. coli* with 17 mm inhibition zone and gram positive bacteria *S. aureus*. 15 mm inhibition zone. The phenolic -OH group present in Clove essential oil was responsible for its antimicrobial activity as the interaction with the polysaccharide, fatty acids and phospholipids of cytoplasmic membrane caused the damage of cellular membrane integrity eventually seepage of cellular contents, reduction of the activity of proton pump consequently resulted death of bacterial cell. The results of present study was in accordance with the literature study of He et al. [22] where the zone of inhibition was (17.8 ± 0.5) mm using clove extract. Another study showed that Clove essential oil had moderate antimicrobial property with zone of inhibition of (8.85 ± 0.17) mm, 10 mm and 13.66 mm [24] certain components in clove essential oil acted on the protein particles of outer membrane of microbe and thereby inactivated [51].

### Evaluation of the hydrogel

#### Encapsulation efficiency

The encapsulation efficiency was determined by quantifying the clove essential oil in the supernatant. The encapsulation efficiency of the hydrogel containing 0.5 g and 1.0 g clove essential oil were 84.65 ± 2.39%, and 72.76 ± 1.95% respectively. The increase in encapsulation efficiency in the hydrogel with 0.5 g of clove essential oil and decrease in the encapsulation efficiency of the hydrogel with 1.0 g of clove essential oil is possibly due to the saturation point of clove essential oil is in between 0.5 g and 1.0 g in gelatin hydrogel [9].

#### Swelling property

The swelling features of the hydrogel were studied. Figure 1(C) confirms the change in the mass of the hydrogel with time. The obtained results show that both the hydrogels reached the swelling equilibrium within 40 min. The hydrogel containing 0.5 g clove essential oil had a higher extent of swelling (34 ± 0.047) % as compared to the hydrogel included 1.0 g clove essential oil (27 ± 0.045) %. Although, clove essential oil had hydrophobic properties, the addition of clove essential oil to the matrix decreased its hydrophilicity, thereby reducing water absorption.

Since gelatin has hydrophilic properties, the control hydrogel had hydrophilic properties. Addition of 0.5 g of clove essential oil to the hydrogel, improved the swelling properties. The cross-linking between clove essential oil and gelatin prevents water loss and ensures that swelling equilibrium is maintained. According to Yang et al. [52] the hydrophilic groups of the polymer chains become ionized when immersed in water, promoting electrostatic repulsion resulting in a higher degree of swelling. The result obtained were consistent with the literature study by Pérez-Córdoba et al. [37] where gelatin/chitosan based films loaded with clove oil droplets in water was used (41.1%).

#### Fourier transform infrared spectroscopy (FTIR)

The crosslinking of hydrogels containing different compositions of clove essential oil (0 g, 0.5 g and 1.0 g) was investigated using FTIR (Fig. 1D). FTIR spectra were taken at 4000 cm^− 1^ to 400 cm^− 1^ range. The results showed that all hydrogels had four major amide groups i.e., amide A, amide B, amide 1 and amide 2. The FTIR spectra showed strong peaks at 3289.91, 1635.80 and 1087.91 cmˉ¹. Absorption peak at 3289.91 cmˉ¹ is due to the formation of -N-H bond and -OH stretching with the amide A group, and the peak at 2926.53 cmˉ¹ is the C-H stretching of the amide B group. Peak at 1635.80 cmˉ¹ was caused by the -C = C and -C = O stretching of the primary and secondary amine N-H bands of amide-1. There are slight differences in peak intensity and width among the three hydrogels. The slight shift in the peak intensity of amide-1 band at 3294.15 cmˉ¹ for the hydrogel containing 0.5 g of clove essential oil and 3287.83 cmˉ¹ for the hydrogel containing 1.0 g of clove essential oil is due to.

the intermolecular interaction between the hydroxyl groups of clove essential oil and gelatin to form amide bands. The absorption peak at 1538.95 cmˉ¹ was recognized for the stretching of the -C-N bond in amide 2, and the peak at 1450 cmˉ¹ was attributed to the stretching of the -C-N bond and the bending of -N-H bond. The absorption peak at 1245.64 cmˉ¹ is the stretching of -C-N bonds and deformation of -N-H bonds. The absorption peaks between 1451.79 cmˉ¹ − 1246.97 cmˉ¹ and between 1245.99 cmˉ¹ and 1450.54 cmˉ¹ of hydrogels containing 0.5 g clove essential oil and 1.0 g clove essential oil are due to the -C = C stretching, -OH bending and -C-O stretching of eugenol molecules. The peak at 1087.91 cmˉ¹ is due to the possible interaction between surfactant and gelatin structure through hydrogen bonding. There was shift in the peak intensity in both hydrogel with 0.5 g clove essential oil at 1090.22 cmˉ¹ and hydrogel with 1.0 g clove essential oil at 1085.41 cmˉ¹. The presence of peaks and shifts in the intensity confirmed the presence of efficient cross-linking [25].

#### X-Ray diffraction

The Fig. 1(E) shows the XRD patterns of blank and clove essential oil -loaded hydrogels. To investigate the crystal structure of these components, measurements were recorded in the 2ϴ range from 5º to 50º.

The blank hydrogel shows the formation of peaks at 2ϴ values of 20º and 8º. These peaks can be attributed to the crystalline nature of gelatin. The crystallinity of gelatin is due to α- helical and triple helical structures of gelatin and is mainly because of the development of interchain hydrogen bond between carbonyl and amine groups. Addition of clove essential oil reduces the crystallinity of the hydrogel. Incorporation of 0.5 g clove essential oil shows a characteristic peak at 2ϴ values of 20º, but with a reduced order as compared to the control hydrogel, when the concentration of clove essential oil was increased to 1.0 g, the peak decreased further. This may be due to the ionic crosslinking between gelatin and clove essential oil, which determines the crystalline properties. This indicated that the encapsulation of clove essential oil into the hydrogel changed the packing structure, thereby reducing its crystallinity, suggesting a partially semicrystalline nature of clove essential oil [11].

#### Scanning electron microscope (SEM)

**Fig. 2:**
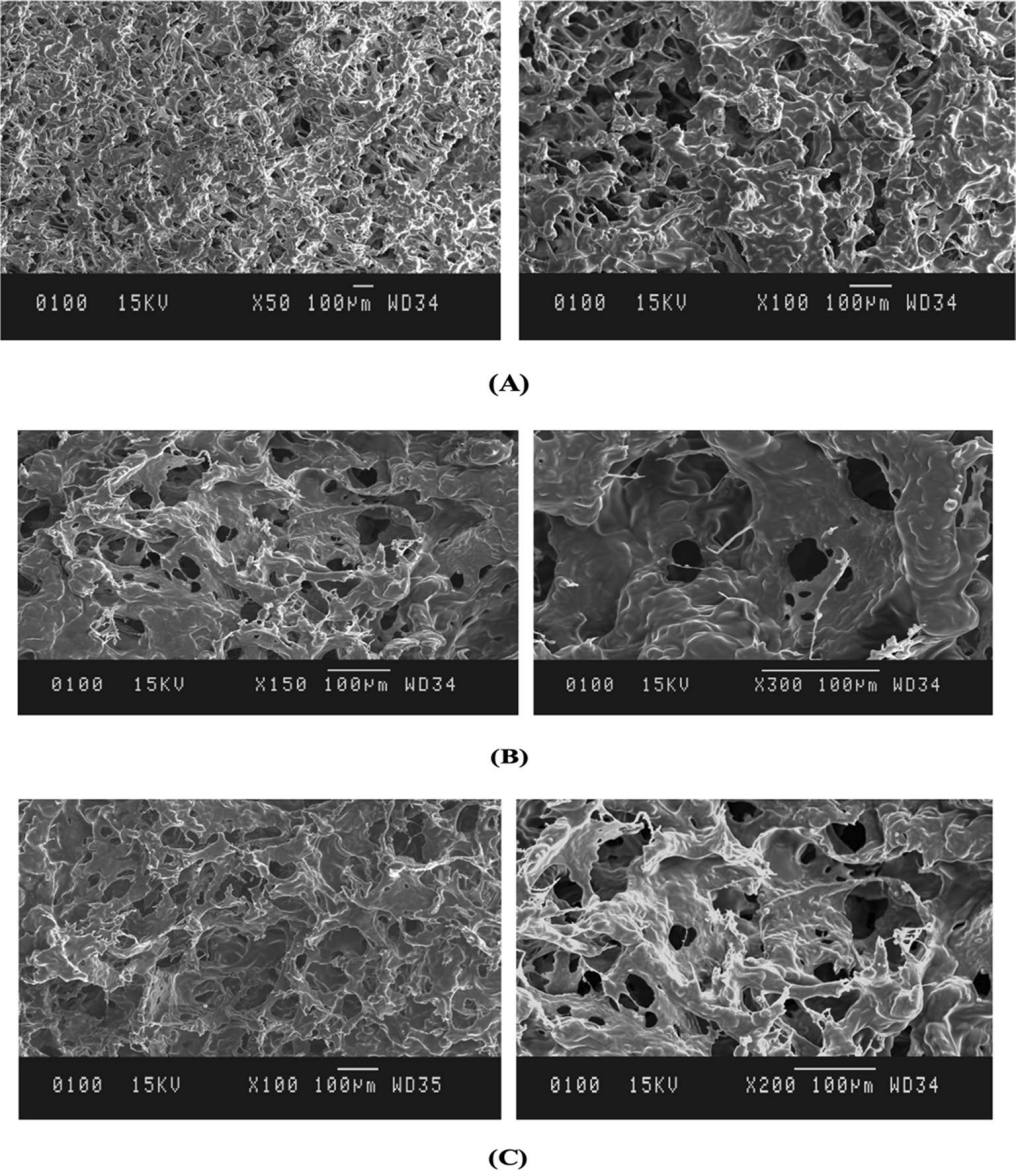
SEM images of **A** Blank hydrogel, **B** Hydrogel with 0.5 g eugenol incorporation and **C** Hydrogel with 1.0 g eugenol incorporation

Morphological studies using SEM were performed to compare blank and clove essential oil encapsulated hydrogels. As shown in the Fig. 2 (A), the blank hydrogel surface is smooth, uniform and homogeneous, with no phase separation, indicating good compatibility. However in Fig. 2 (B) and 2 (C), when clove essential oil was incorporated into the hydrogel, the pores become visible and its microstructure changed. When 0.5 g of clove essential oil was incorporated into the hydrogel, the SEM images showed an irregular and rough surface forming gelatin aggregates, which gradually formed a spherical structure, resulting in the appearance of pores within it when the concentration of clove essential oil in the hydrogel increased to 1.0 g, This can be attributed to the ionic crosslinking between gelatin and clove essential oil. Therefore, it was observed that when an increased clove essential oil concentration was incorporated into the hydrogel, the ionic cross-linking between clove essential oil and gelatin resulted in denser and thicker pore walls [48].

#### Contact angle

**Fig. 3:**
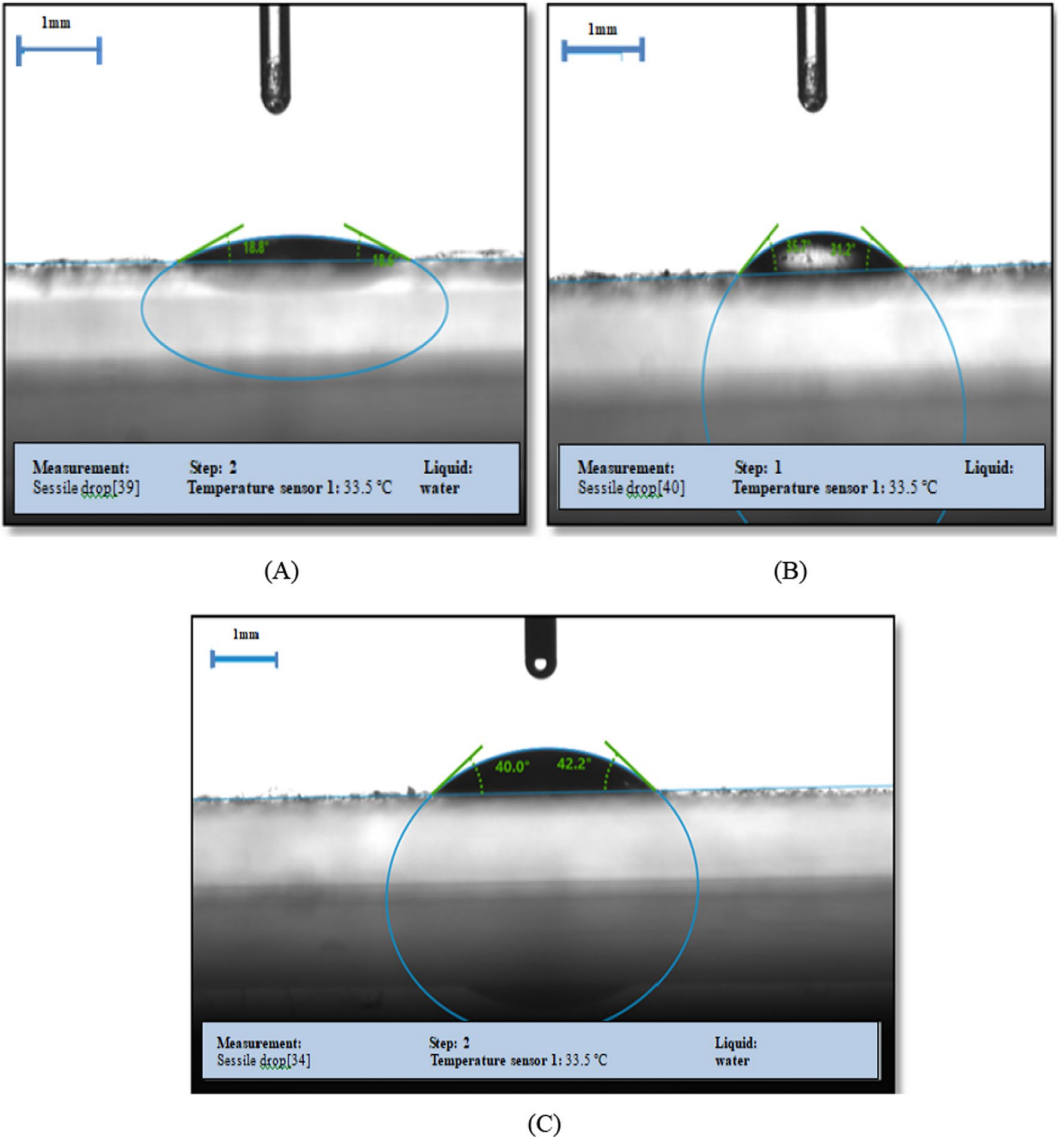
Contact angle measurement of hydrogels **A** Control hydrogel **B** Hydrogel containing 0.5 g eugenol **C** Hydrogel containing 1 g eugenol

Contact angle was performed to determine the hydrophilicity and hydrophobicity of hydrogels. The Fig. 3 demonstrates the images of contact angle of the hydrogel with or without the presence of clove essential oil. In Fig. 3 (A), the contact angle of hydrogel is 18.8 ± 0.42º, signifying that it has a more hydrophilic nature with gelatin. In Fig. 3 (B), addition of 0.5 g of clove essential oil to the hydrogel increases the angle to 35.7 ± 0.37º. In Fig. 3 (C), the addition of 1.0 g of clove essential oil increased the contact angle to 40.0 ± 0.36º, indicating that it is due to the hydrophobic character of the clove essential oil. Gelatin is a hydrophilic component with a contact angle of 18.8 ± 0.42º, so its use as a food packaging material has been limited. However, the addition of clove essential oil improved the hydrophobicity of gelatin hydrogels and improved their use as active packaging materials [41]. The present study on the surface wettability of hydrogels was similar to the literature studies by Gupta and Ghoshal [19], where contact angle of pure green gram hydrogel was 40.75° and in curcumin incorporated hydrogel the angle increases to 49.35°.

#### Color

Hunter color values showed that the clove essential oil loaded hydrogel had a distinctive reddish color at a given a* value. The brightness of the hydrogel was confirmed by the L* value. The yellowish b* effect of the hydrogel is due to the presence of pale yellow pure clove essential oil. Comparable results were obtained by Wang et al. [49]. The L* value was 57.71 ± 0.029, confirming the lightness of the hydrogel, the value of a* parameter was 22.01 ± 0.180, confirming the redness of the hydrogel. The values of the b* parameter and **∆**E were 13.92 ± 0.213 and 62.858 ± 0.140 respectively confirmed the yellowish effect of the hydrogel.

#### Antioxidant activity

**Fig. 4:**
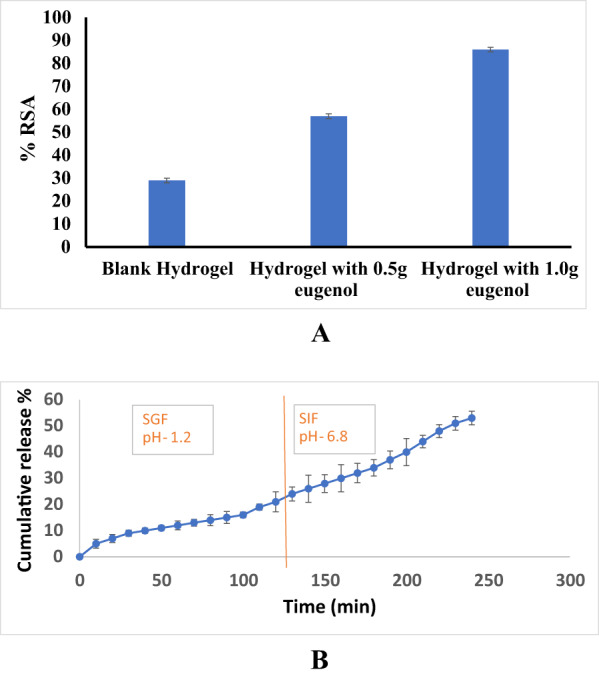
**A** DPPH Inhibition % of blank and eugenol loaded hydrogel. **B** In vitro drug release of eugenol loaded hydrogel

The antioxidant activity of the hydrogels was evaluated using 0.1 mM of DPPH in methanol at 517 nm. The Fig. 4(A) shows that the scavenging activities of the clove essential oil loaded hydrogels are 57 ± 0.026% and 86 ± 0.035% for 0.5 and 1.0 mg clove essential oil respectively, which are higher than the control hydrogel sample with 29% activity. Therefore, clove essential oil has good antioxidant properties, and the antioxidant activity of hydrogels increased significantly as the concentration of clove essential oil incorporated into the hydrogels increased. The phenolic hydroxyl group present in clove essential oil has the ability to scavenge free radicals by donating hydrogen atoms or electrons, thereby terminating the peroxide chain reaction mechanism, and is responsible for its potential antioxidant activity [31], it was observed that when the clove essential oil concentration in gelatin nanofibers increased from 2 mg/g to 4 mg/g, the radical scavenging activity increased from 17.83% to 43.80%.

#### In vitro drug release

The intention of the in-vitro drug release study was to establish the release rate of the nutraceutical from the carrier and to test how far the nutraceutical can penetrate in-vitro through the membrane. Figure 4(B) exhibits the release of clove essential oil from the hydrogel under simulated gastrointestinal circumstance and simulated intestinal conditions. The release of clove essential oil in the simulated gastric fluid (SGF) at pH 1.2 was observed to be initially slow for 2 h. Drug release after 10 min was 5% and after 120 min was 21%. However, the release rate of clove essential oil in simulated intestinal fluid (SIF) started to increase slowly at pH 6.8, with release of 24% after 130 min and release of 53% after 240 min. The slow release of clove essential oil at pH 1.2 may be due to the insufficient swelling rate of the simulated gastric fluid. The increased release of clove essential oil at pH 6.8 may be due to the fast dissolution rate of the clove essential oil loaded gelatin hydrogel. The obtained results were consistent with the literature study by Shao et al. [40], where the release of clove essential oil initially shows a rapid release, followed by a controlled slow release until a plateau i.e. 55% is achieved. Nagaraju et al. [35] was observed that eugenol release was 28% at pH 2.4 in the stomach, compared to 60% at pH 7.4 in the intestine.

### Coating of hydrogel in fresh apple

#### Weight loss

**Fig. 5:**
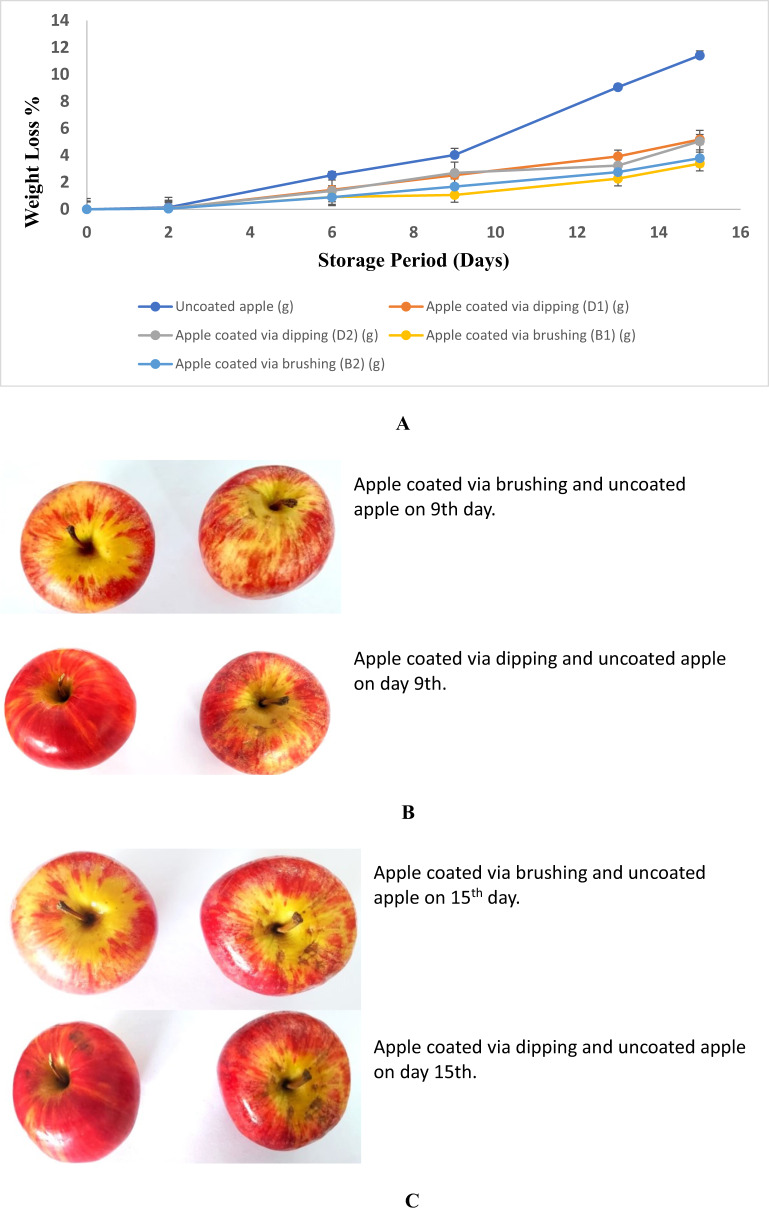
**A** Weight loss % of apple samples. **B** Comparison of apple coated via brushing and dipping method with the uncoated on day 9. **C** Comparison of apple coated via brushing and dipping method with the uncoated on day 15

As shown in Fig. 5(A), the loss of weight in apple was due to transpiration. Moisture transfer from apples to the environment is thought to be the main cause of weight loss during storage [17, 18]. Gelatin hydrogels loaded with clove essential oil acted as a moisture barrier, reduce transpiration eventually reduce weight loss. The weight loss was observed in uncoated and coated apples both with hydrogel during the storage period of 0, 2, 6, 9, 13 and 15 days. Since clove essential oil loaded gelatin hydrogel was applied as active packaging material, the results showed that the weight loss of uncoated apple was higher (11.41 ± 0.028%) during the storage period than the apples coated with brushing (3.39 ± 0.021%, 3.79 ± 0.039%) and dipping method (5.05 ± 0.039%, 5.18 ± 0.025%). Therefore, it was observed that the hydrogel coating effectively reduced the weight loss of apple indicating that the hydrogel had greater water holding capacity and it acted as a moisture barrier. Similar result was also seen in research study of Chen et al. [8] where the clove essential oil delayed the weight loss, values on the days of 6th and 9th during storage by 1.78 ± 0.25% and 2.98 ± 0.27% respectively, which indicates that hydrogel coated via brushing method was more effective than the dipping method.

#### Sensory evaluation of apple

Table 3 shows the result of the sensory evaluation of both non-hydrogel coated and hydrogel coated apples [14].

**Table 3:** Sensory evaluation (overall acceptability) of Apple samples during storage period

Sample	0 days	2 days	6 days	9 days	13 days	15 days
Uncoated apple	5.0 ± 0.0	4.6 ± 0.1	3.6 ± 0.3	2.9 ± 0.4	2.1 ± 0.2	1.9 ± 0.5
Dipped apple	5.0 ± 0.0	4.8 ± 0.2	4.5 ± 0.4	4.2 ± 0.1	4.0 ± 0.4	3.9 ± 0.2
Brushed apple	5.0 ± 0.0	4.9 ± 0.3	4.8 ± 0.1	4.4 ± 0.3	4.2 ± 0.1	4.1 ± 0.1

On first day of storage each apple sample has a fresh, pleasant taste, aroma, and color. During the storage period, hydrogel-coated and uncoated apples were observed to remain fresh and maintain good sensory properties for up to 9 days (Fig. 5B). But after 9 days, the apples gradually began to decompose, the consistency became soft and an unpleasant odour gradually appeared. Therefore, the initial sensory score for all apple samples was 5. After 15 days of storage shown in Fig. 5(C), the various sensory scores of the uncoated apple sample decreased to 1.9, while the sensory score of the coated apple using the dipping method remained at 3.9. Apples coated using the brushing method remained at 4.1. Therefore, the results suggest that the hydrogel coating delayed the sensory deterioration of the apple samples. Wang et al. [50] obtained that the chitosan coated sample after 15 days has shown reduction in the sensory score value to 2.8–3.4 but clove essential oil loaded coating samples values were still greater than 3.6 [21].

#### Firmness of apple

The firmness of the uncoated apples decreased about 12.3 to 7 g cmˉ^2^ over the storage period, whereas the hardness of the coated apples using the dipping and brushing remained about 11 g cmˉ^2^. Softening of uncoated apples occurred mainly through enzymatic reaction of the cell wall. However, the hydrogel coating of apples provided a mildly acidic environment, which reduced the activity of the enzymatic hydrolysis and extended the shelf life by several days. Similar results were obtained in a literature study by Li et al. [32], they demonstrated that as content in the films were increased, the firmness loss has decreased. This may indicate the ability to delay the cell breakdown.

## Conclusion

In this study, clove essential oil was successfully extracted from clove buds using maceration and Soxhlet extraction methods. The maximum yield obtained using the Soxhlet method was 44%. Encapsulation of clove essential oil into hydrogels proved efficient, imparting antibacterial and antioxidant properties to the applied fruit. Clove essential oil exhibited excellent antibacterial properties against *E. coli* and S. *aureus* strains, with a minimum inhibition zone of 17 mm and 15 mm. The coated apple samples demonstrated good sensory attributes and firmness while experiencing less weight loss. The hydrogel coating on the surface of the apples served as a moisture barrier, reducing weight loss and slowing spoilage, ensuring the retention of their sensory properties. Furthermore, the gelatin used to manufacture the hydrogel is biocompatible and edible, so any residues left behind will not have any adverse effects. Finally, the results obtained in this study demonstrate the effective application of hydrogels to extend the shelf life of apples.

## Data Availability

The supporting data of this study are available on request from the corresponding author. The data are not publicly available due to privacy or ethical restrictions.
